# 

**Published:** 1884-05

**Authors:** 


### CONTENTS.

ORIGINAL COMMUNICATIONS—

Mercury. By J. S. Todd, M. D., Atlanta, Ga. . . . . . . . . . 129

Most Efficient Treatment of Scalp Wounds. By J. McF. Gaston, M. D., Atlanta, Ga. . . . . . . . . . . . . . . . . . . . . 143

Episcleritis with Degeneration of Iris–Iridectomy for Restoration of Sight. By Chas. W. Hickman, M. D., Augusta, Ga. . 148

SELECTIONS—

Punishment a Factor of Inebriety and Insanity. By T. D. Crothers, M. D., Hartford, Conn. . . . . . . . . . . . . . . . . . . 150
Tonsillitis. By T. A. DeBlois, M. D. . . . . . . . . . . . . . . 158

SOCIETY PROCEEDINGS—

The Medical Association of Georgia. . . . . . . . . . . . . . 164

EDITORIAL—

Medical Association of Georgia. . . . . . . . . . . . . . . 187

Books Received. . . . . . . . . . . . . . . . . . . . . . . 188

Our Portrait Gallery. . . . . . . . . . . . . . . . . . . . . 189

Our Advertisers. . . . . . . . . . . . . . . . . . . . . . . 190

NOTES—

Testis in Perineo, 147; How to take a Pill, 149; Rule for Reducing Dislocations of the Hip Joint, 157; Tubal Fœtation, 163; American Medical Association, 185; The Sims Memorial Fund, 185; Florida State Medical Association, 186; Facial Erysipelas, 191; A Toothache Remedy, 192; Death from Ether, 192; Items, 192.

### NOTICE.

